# A public health approach to understanding and preventing violent radicalization

**DOI:** 10.1186/1741-7015-10-16

**Published:** 2012-02-14

**Authors:** Kamaldeep S Bhui, Madelyn H Hicks, Myrna Lashley, Edgar Jones

**Affiliations:** 1 Wolfson Institute of Preventive Medicine, Queen Mary, University of London, London, UK; 2 Health Service and Population Research, Institute of Psychiatry, King's College London, London, UK; 3 Culture & Mental Health Unit, Lady Davis Institute, Jewish General Hospital, Montreal, Quebec, Canada; 4 King's Centre for Military Health Studies at the Institute of Psychiatry, King's College London, London, UK

## Abstract

**Background:**

Very recent acts of terrorism in the UK were perpetrated by 'homegrown', well educated young people, rather than by foreign Islamist groups; consequently, a process of violent radicalization was proposed to explain how ordinary people were recruited and persuaded to sacrifice their lives.

**Discussion:**

Counterterrorism approaches grounded in the criminal justice system have not prevented violent radicalization. Indeed there is some evidence that these approaches may have encouraged membership of radical groups by not recognizing Muslim communities as allies, citizens, victims of terrorism, and victims of discrimination, but only as suspect communities who were then further alienated. Informed by public health research and practice, a new approach is proposed to target populations vulnerable to recruitment, rather than rely only on research of well known terrorist groups and individual perpetrators of terrorist acts.

**Conclusions:**

This paper proposes public health research and practice to guard against violent radicalization.

## Background

In this paper we propose that public health research and practice can inform preventive strategies and interventions against violent radicalization. The term 'radical' can mean 'politically subversive', 'creative', or 'extreme', without necessarily being illegal, criminal, or a threat to society. However, recent research and policies that address terrorism have used a more specific term, that of violent radicalization. Violent radicalization is conceptualized as a social and psychological process, often facilitated by recruitment and training, by which an individual becomes increasingly committed to politically motivated violence, especially against civilians (See Table 1 for definitions) [1,2].

**Table 1:** Definitions

Term	Definition
Radicalization	The social and psychological process of increasing commitment to extremist political or religious ideology [1]
Violent radicalization	The social and psychological process of increased and focused radicalization through involvement with a violent non-state movement. Phases are (a) becoming involved with a terrorism group and (b) remaining involved in or engaged with in terrorist activity [1]
Terrorism	Participation in politically motivated violence or threat of violence, especially against civilians, with the intent to instill widespread fear [2]
Disengagement	The process whereby an individual has a change in role or function associated with reduction of violent participation, either enforced (for example, imprisonment) or due to psychological factors (for example, disillusionment) [1]
Deradicalization	The social and psychological process, or intervention, by which commitment to, and involvement in, violent radicalization is reduced to the extent that the individual is no longer at risk of involvement in politically motivated violent activity [1]
Counter-radicalization	Interventions to prevent social or political radicalization, to prevent violent radicalization, or to disrupt involvement in terrorism of those already radicalized [1]

The motivations and ideology of groups that undergo violent radicalization are often specific to political, historical, social and cultural contexts that can shape their extremist actions and the accompanying political rhetoric [3,4]. Since 9/11, the term 'violent radicalization' has been applied mostly to extreme Islamist groups as they have claimed responsibility for the majority of recent terrorist attacks across the world [4,5]. The public health impact of terrorist acts arising from violent radicalization includes direct consequences such as deaths, physical injuries and psychological or mental injuries. The 9/11 attacks in 2001 killed nearly 3,000 individuals. The London 7/7 bombings in 2005 killed 52 and injured close to 700. Between 2003 and 2010, suicide bombers in Iraq killed 12,284 Iraqi civilians and injured a further 30,644. The groups that claim responsibility selectively invoke religious rhetoric to justify politically motivated violence but they do not generally represent Muslim populations or Islamic Fundamentalism [1,6-9]. Thus, a public health response to political violence [10] will need to consider that the majority of the victims globally are in fact Muslim civilians [5,11]. However, psychological sequelae after 9/11 were found more generally among all populations who had experienced personal losses of friends, relatives, and jobs [12]. Other direct effects include bereavements, loss of employment, economic damage, fear and distress. Indirect effects include a diversion of resources to tackle terrorism and rebuilding communities, social divisions within communities along religious lines, restrictive counterterrorism actions and inconvenience, discrimination against those perceived to be associated with terrorism, and of course the sending of and potential loss of troops to international conflicts where terrorist related activity is thought to arise. These consequences can also be mapped using public health approaches, and are not fully reviewed in this paper, which is devoted to understanding violent radicalization to mount effective strategies to reduce the likelihood of future incidents.

Until the 7/7 London bombings, a widely accepted perspective in the UK and the US was that terrorist threats originated from foreign radical Islamists, as in the 9/11 attacks [6,13]. After the 7/7 bombings in London, concerns in the UK shifted towards reducing the threat from a 'third wave' of 'homegrown' terrorists who were recruited and radicalized within the UK; similar responses were found in Canada and US and other European countries in response to similar incidents in those countries [6,13,14]. For example, Crone and Harrow studied 228 individuals who participated in 65 Islamist terrorist plots or attacks in the West between 1993 and 2008 [13]. They found that the majority of these plots involved people who had spent their formative years in the West and seemed to operate independently of any terrorist organization [13]; although, in reality, the majority involved some organized recruitment, training, or financing by external terrorist organizations [13]. These trends pose a challenge that requires a new response including population level research of putative risk and protective factors for violent radicalization (Table 2), rather than relying only on criminal justice system evidence from convicted terrorists. This paper discusses these risk and protective factors (Table 2), and proposes a public health model of research and prevention [10].

**Table 2:** Putative risk and protective factors

Factor	Description
Risk factors	Young people facing transitions: education, place, family, religion and so on
	Cognitive and social openings to new influences
	Social isolation and exclusion
	Grievances about discrimination that may be personal, related to unfair treatment at work, access to health care or about other inequalities in society
	Unemployment
	Migrant status and experiences before and after immigration
	International conflict that is considered unjust against a group with which individual identifies on religious, national or cultural grounds
	Perceived threat to family and cultural group
	Marginalized and traditional cultural identities
	Discrimination thought to explain group inequalities in health and social status and access to wealth
	Not able to negotiate needs and protest through non-violent and democratic means
	Contact with influential or charismatic leaders who justify terrorism (for example, in prisons, or in schools or universities)
Protective factors	Social support
	Social cohesion
	Social capital and trust in institutions
	Feeling of safety and security in neighborhood
	Integrated cultural identity
	Employment success
	Access to democratic means for negotiating needs and opinions
	Access to critical religious leadership that can moderate and inform on legitimate religious perspectives

## Discussion

### The limitations of criminal justice approaches

Efforts to understand the motivations of terrorists and the pathways leading to violent radicalization have largely adopted a criminal justice system framework. In the UK, for example, the PREVENT strategy sought to gather intelligence and facilitate the prosecution of anyone thought to be associated with terrorism, guilty of terrorist acts, or in possession of materials that might assist terrorism. One consequence is an ethical dilemma for many researchers trying to investigate terrorism and violent radicalization as confidentiality cannot be offered to research subjects if they reveal information about possible terrorist acts. Indeed, researchers themselves can be liable to prosecution if they fail to disclose information to the authorities, or if any information they acquire later transpires to be of importance in identifying terrorists. Furthermore, researchers may themselves come under suspicion if, during their investigations, they examine websites or propaganda used by terrorists [15,16]. These considerations can restrict the capacity of researchers seeking to understand how sympathies for violent radicals and terrorists emerge in populations, and how vulnerabilities are exploited in recruitment processes.

The criminal justice system approach assumes that the legal system can effectively deal with crimes irrespective of their origins and contexts, and that terrorism can be prevented by criminal intelligence and a tailored judicial system rather than through engagement with other bodies of theory and practice. By contrast, we argue that epidemiology, psychology, sociology and other behavioral sciences can contribute important data towards prevention strategies, which have been used in public health programs to address violence. Such alternatives do not condone the conduct of terrorists, but aim to investigate the wider determinants of recruitment in vulnerable populations and to identify the pathways to radicalization, rather than simply considering evidence from convicted terrorists and only from criminal justice analysis [3,9]. A wider analysis is necessary because, as Atran sets out, there is no shortage of volunteers to join the ranks of martyrs in the Middle East, but the situation in the UK and in the US and Canada is less well known and the process of violent radicalization and its impacts in populations are not fully understood [3,9].

An exclusive emphasis on a criminal justice system strategy may impede prevention. For example, counterterrorism initiatives by the British government stigmatized and alienated Muslim communities in the UK by treating them as a conspicuous religious group that was under suspicion rather than as allies in a preventive strategy [17,18]. Counterterrorism policies, however well intentioned, did not inspire confidence or attract support as they were seen as unjust and so they actually damaged social cohesion by isolating a religious group [17,18]. Social cohesion is the ability of a society to be inclusive of all cultural and social groups, so that they work co-operatively. It has many benefits including everyone realizing their potential (human capital), and greater social capital (a form of social wealth on which society draws), and greater social support. Each of these has been linked with better population health and more equal and just societies, and also with less violent crime, especially in US studies. Thus the support and engagement of Muslim minorities is important to counter the arguments of those who perceive the authorities as unjust and discriminatory against Muslims. The experience from Canada is consistent with findings in the UK; that not working with and through the population as a whole will undermine counterterrorist strategies. In Canada, a helpful public health intervention was the setting up of a crosscultural round table on security. This provides a forum for ongoing discussion with community involvement and support, and it links policies across other arenas of importance such as education, health, and employment; the round table also assists in determining when counterterrorism should focus on violent radicalization linked with Islamist groups or all extremist groups, given that not all terrorist attacks are claimed by or linked to Islamist groups [19]. The initiative in Canada provides an example of the importance of complementing the criminal justice approach with a public health approach to terrorism and violent radicalization.

### The public health approach

#### Definition and principles of public health

**Figure 1:**
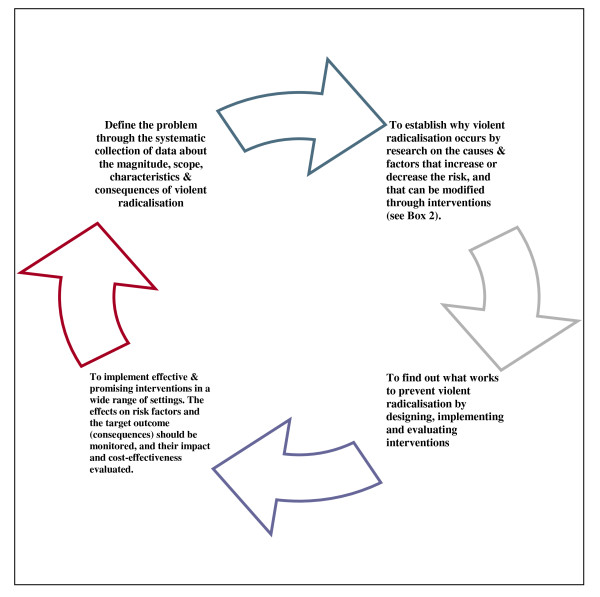
**A public health approach to preventing violent radicalization**.

Public health, as defined by the UK Faculty of Public Health, is 'The science and art of promoting and protecting health and well-being, preventing ill health and prolonging life through the organized efforts of society' [20]. Public health interventions are predominantly population based. These emphasize collective responsibility for health, health protection, and disease prevention, and recognize underlying socioeconomic and wider determinants of health and disease. The approach emphasizes partnerships with all those who contribute to the health of the population. Health improvement involves attention to education, inequalities, housing, employment, lifestyles, family and community, and surveillance systems. In prevention in public health, population level reductions in characteristics (or behaviors) that carry a small individual risk for a particular illness lead to greater reductions in the overall prevalence of that illness, when compared with interventions on very few people who are identified as carrying a very high risk. This approach has been applied to violence prevention so reducing the mean levels of any particular risk factor in the population [21]. For example, the World Health Organization's Violence Prevention Alliance provides a public health framework to investigate and understand the causes and consequences of violence [21]. Similar approaches are used by the Centers for Disease Control in the USA (see http://www.cdc.gov/ViolencePrevention/overview/publichealthapproach.html). However, extending such work to tackle violent radicalization and terrorism is a natural step but rare. Therefore, a similar approach can be applied to violent radicalization (see Figure 1).

#### A public health approach to tackle violent radicalization

Public health approaches are already applied in bioterrorism and street violence (for example, knife crime injuries) [2]; but in most countries, including the UK, public health agencies have not yet taken up violent radicalization as a focus of research and intervention. A surveillance system can monitor deaths and injuries due to terrorist actions, but might also include other risk and protective factors for violent radicalization. For example, risk and protective factors set out in Table 2, and perceived discrimination in the population as a whole or amongst specific segments of the population; trust in authorities and in their counterterrorism approaches; perceived or real economic inequalities patterned by ethnicity or religious groups; and international conflict in which the authorities appear to be biased or unfair towards a specific migrant, religious or ethnic group.

Global databases on violent radicalization and terrorism are being compiled but are generally restricted to event counts, and are also subject to many potential sources of bias and confounding. Dugan *et al*. describe the development of the first open source database on global terrorist incidents [22] and discuss the limitations of these data: (1) most terrorists are not legally processed for terrorist offences but for other related offences, and most terrorism data are outside the realm of domestic criminal justice systems. (2) Data collected are also subject to politically determined priorities given much of it is collected by government agencies; and most data sources are not routinely available for researchers. (3) Most research is based on secondary or tertiary source data that do not meet academic standards.

An implication of these findings is that terrorist acts are classified as being distinct from criminal acts. Whilst terrorism is dealt with by criminal justice agencies, feedback and evaluation of programs is not easy to undertake. Terrorism databases do not include measures of risk factors for violent crime and violent radicalization, which are more common in populations; nor do these databases link with other potentially important variables (which we later discuss). Only by collecting a fuller range of relevant variables (shown in Table 2 and new variables identified through research in Figure 1) can a surveillance system generate evidence about patterns of incidents and the correlates at individual and population levels. More research is required on relevant risk and protective factors for violent radicalization.

As well as measuring terrorist-related direct deaths and injuries [21,23], it is important to study the psychological effects of terrorist acts on public mental health and on social cohesion [12]. Understanding psychological trauma suffered by witnesses and casualties can inform how to respond to terrorism in ways that encourage people to be prepared, vigilant and informed without living in fear and maximizing recovery from psychological problems [12]. Routine health surveys and censuses might ask about aspects of violent radicalization and group cohesion as relevant health and social issues, and not only as a criminal justice issue. The International Classification of Diseases and Centre for Disease Control now has codes for deaths and injuries from terrorist acts; this offers a system of monitoring closely aligned to other public health surveillance programs. Its use may offer one mechanism to look objectively at global terrorism-related deaths and circumstances in which they occur [23]. This should make the most severe effects of violent radicalization (deaths and injuries) easier to monitor and relate these to other contextual and demographic data at both ecological and local levels.

Other important variables include fears about personal safety when under suspicion and fears of being wrongly accused of being a terrorist; these also can undermine social cohesion and social capital, both of which exercise important influences on the health and well-being of populations and on risks of community violence [18,24]. We do not currently measure population indices of these, or of grievance, intergroup prejudice, or international conflicts, all of which have been implicated in terrorist activities. Some health-related risk factors may also be risk factors for violent radicalization. For example, discrimination is associated with poorer health [25,26]; and a recent global analysis of discrimination experienced by minorities shows economic discrimination is closely related to domestic terrorism [27], and this may increase the risks of international terrorism [28]. Discrimination can also be a marker of social isolation in society, social exclusion, and unemployment. These conditions make young people vulnerable to extremist influences and ideologies while weakening their ties with socially inclusive influences [9]. A weakening of ties to healthy influences can occur during transitions such as migration, maturation during adolescence, changes in schools or universities, changes of employment, geographical mobility and religious conversions. McCauley and Moskalenko describe how isolation can lead to radicalization through a process of unfreezing of links and isolation from healthier and safer networks, giving rise to openings to new influences including gangs, cults and other ideologically driven groups that provide powerful feelings of belonging and loyalty and positive identity and self-esteem [4]. Maintaining a positive regard for the authorities and healthier influences appears to be an important protective factor against violent radicalization in the first place; therefore, nurturing strong identifications with healthier influences is likely to be as important as minimizing contact with gangs, cults, and networks associated with violent radicalization.

### Researching complex pathways to violent radicalization

Drawing on the approach taken by Weine and colleagues to Somali-American youth [29] and Horgan's pathways to violent radicalization [1], we propose that a public health approach needs to be applied at the population level to engage a larger proportion of the population at risk of violent radicalization. This recognizes that very few people proceed all the way to committing a terrorist act and that many influences that make this more likely are potentially modifiable. This approach requires an understanding of individuals' and groups' biographies, identities and stories, the cultural influences on socialization and successful resettlement, and public and community support for counter-radicalization. We propose that this will yield greater gains than current approaches that attempt to target only those already planning or committing terrorist acts, or those in contact with the criminal justice system, neglecting the wider population base from which terrorists are recruited and the networks with which they are associated. The proposed approach also decreases the risk of stigmatization associated with profiling, for which there is little empirical evidence of predictive accuracy. Thus, pathways to violent radicalization can be better understood if public health research investigates promising new variables from the social and behavioral sciences such as social inclusion, exclusion, cultural identity and acculturation, stigma, discrimination, and political engagement [10,30]. It will be important to test the predictive validity of these putative risk and protective factors. For example, there is value in measuring popular sympathies for violent radicalization compared with levels of political engagement in the same population, and to studying their relative effects on health and on violent and non-violent protest.

An example of how these influences might interact is found in the case of violent radicalization of youth in Somali-American communities [29]. Between 2007 and 2008, an estimated 18 Somali-American adolescent boys and young men left their homes in Minneapolis, without knowledge of their friends or family, to join militant training camps run by the Al Shabaab, an extremist group in Somalia. Among them was 27-year-old Shirwa Ahmed, who became the first known suicide bomber with US citizenship when he detonated his car bomb in an attack on a government office in Somalia. Taking a public health and psychosocial approach, these Somali-American youth may have been vulnerable to the appeal of violent radical ideas because of war-related displacement; fragmentation of family and community structures when moving from Somalia to refugee camps, then to settlement in the US; feeling trapped between dissonant acculturative identities with insufficient adult guidance from the immediate community and family; and identification with a 'warrior' role adopted by some Somalis during the Ethiopian invasion of Somalia [29]. Identification of these risks for violent radicalization informed the development of specific community appropriate interventions [29].

This case study shows that violent radicalization can be an outcome of a complex interaction between social, political, cultural, historical, and interpersonal factors [1,5,6,30-33]. As a result, research will need to include religious variables, measures of conflict, measures of acculturation and cultural identity, human capital and migration-related factors. It is known that one outcome of acculturation can be a more traditional identity, which in some instances can be a form of protest against a dominant culture that is perceived as unjust, or a method to preserve familiar cultural practices and roles in the face of uncertainty. Therefore, studies to verify the risk factors of importance in violent radicalization will require innovative methods to measure cultural integration and citizenship, fair access to health, wealth and material resources, alongside measures of sympathies for, or condemnation of, different protest groups as well as terrorist groups.

### Early prevention of violent radicalization

Some studies suggest that violently radicalized individuals are no more likely to have histories of deprivation, unemployment, criminality or poverty than others in their wider community [31,32]. If anything, they are better educated, slightly younger and slightly more likely to be migrants than the general population [31,32]. At the moment, it is difficult to target preventive interventions at individuals who plot terrorist attacks because they are not particularly identifiable by demographic or personal characteristics, or by psychopathology, and there is often no formal membership structure or hierarchy among violent radicalized groups [1,6,15,30-32]. However, evidence suggests that a useful focus is on young people who are vulnerable to radicalizing influences because of isolation or marginalization, particularly as they are likely to be accessible to interventions while in full time education during adolescence and young adulthood when identity-related psychological and social transitions are common [4].

Given what is known so far about homegrown terrorism, preventive interventions should focus on how negotiations of personal identity, social exclusion and marginalization can generate grievances that are not processed through political engagement, nor through democratic non-violent negotiations [3,11,9]. If grievances emerge from experiences of discrimination and among people with particular cultural identities (for example, traditional or marginalized) then, like the plethora of interventions to reduce discrimination and improve integration, violent radicalization should be amenable to a public health approach. Research and practice would need to encompass three elements: (1) the ongoing process of changing personal and community affiliations in relation to transnational ideologies, identities, histories, events, traditions and cultures in order to promote integration which is known to yield health benefits through social benefits; (2) social, immigration, human rights and public health policies that can promote trust and social and political engagement; and (3) violence prevention and disaster management that involves all people in a preventive strategy.

Educational policy may also be important for a variety of reasons. Focusing counter-radicalization interventions on secondary schools and centers of higher learning could reach a wide social group at a formative stage when many young people explore modes of engaging with 'radical' and alternative perspectives, and many grapple with identity issues. Young people face many transitions in friendships, identity, and in their homes. This is a crucial time of flux when individuals are beginning to take a view on international events and on their own sociopolitical identity. Violently radicalized individuals tend to be younger and better educated than many in their communities. Because terrorist recruiters target educational environments and young people, counter-radicalization interventions located in education may be very well placed to protect individuals from induction into violent radicalization [10,15,32]. The notion of teaching healthy relationships, healthy lifestyles, citizenship and good character in schools is not a new one. However, adaptations may need to be made to accommodate the historical accounts and intergenerationally transmitted narratives that vulnerable people absorb in their homes and in their culturally defined communities [33,34]. Stories told within communities and homes can enhance feelings of prejudice and intergroup conflicts by narrating and reactivating traumas associated with monstrous acts by others; all of these can then position the identity of the child, family and community, along ethnic, religious, cultural or demographics characteristics [34]. These narratives may differ from official historical accounts that are taught and may be used by terrorist groups to foster violent radicalization [33]. Thus schools might need to address these concurrent, seemingly incompatible, narratives while taking care to nurture pride in the histories of marginalized communities. It is also a responsibility for teachers and role models to ensure that their own prejudices, assumptions, and family stories are not automatically transmitted to the next generation. Because highly educated terrorists are more likely to succeed [3,31] and kill more people when they do [8], decreasing the violent radicalization of young people in higher education may yield significant benefits in public protection. Supportive evidence for such an approach is found from studies in the Middle East, which show that higher educational status is associated with greater sympathy for violent radicalization and terrorist movements [3].

Analyses suggest that growing up in politically radicalized communities is an important but insufficient factor for violent radicalization [35-38]. Interventions that reduce sympathies for violent radicalization may improve social cohesion and non-violent political engagement and vice versa. It is important to find ways of preventing political moderates or the politically uncommitted in a community from developing sympathies for violent extremist ideologies based on perceived attacks on their religion or identity group [3,10,37]. Engaging people from all political, religious, and demographic backgrounds is essential to ensure public support for social cohesion and public safety. This also has wider health benefits of cohesive communities with greater social capital and fair access to health, wealth and material resources.

## Conclusions

Applying a public health approach to the prevention of violent radicalization in the population will include a search for risk and protective markers that can be a focus of interventions to minimize recruitment to violent radicalization. A public health approach has the potential to foster social inclusion and social justice in communities that feel threatened by terrorism, to help destigmatize 'suspect communities', and to identify and address common issues of grievance or marginalization. In addition, a public health approach can facilitate the identification of factors to protect individuals from induction into violent ideologies during critical developmental periods. There will also be wider health and social benefits where the risk factors for violent radicalization are also risk factors for violence and poor health in general and social inequalities.

## Competing interests

The authors declare that they have no competing interests.

## Authors' contributions

All authors discussed the evidence and contributed to consecutive versions of the paper. KB provided the first draft and the finalized draft based on comments from all authors and discussion between authors. KB's work involved cultural identity, acculturation and social inclusion, including religious factors and discrimination, and impacts on mental health and personality. MHH contributed ideas and research from war studies and studies of terrorism and its public health impact. ML is involved in Canadian counterterrorism policies and public safety, and chairs the Cross-Cultural roundtable on Security in Canada. EJ's contributed experience is in historical analyses of terrorism and the psychological effects of conflict. All drafts were discussed and edited by all authors each contributing original ideas and views.

## Pre-publication history

The pre-publication history for this paper can be accessed here:

http://www.biomedcentral.com/1741-7015/10/16/prepub
